# The African Fever

**Published:** 1846-01

**Authors:** 


*The African Fever.*—We have heard of no fresh cases of this disease since our last. Although some of the crew of the Growler, stationed at Woolwich, had been attacked by the fever, it had not assumed a malignant character, and all restrictions on the vessel have in consequence been removed. The last accounts of the crew of the Eclair represent that six of the men were labouring under the fever, two having it in a very severe form. One man was suffering from a second attack. The disease has been most fatal among the crew of this vessel. Out of 145 officers and men, her full complement, 123 have been attacked by the fever since April last, and 65 have died, representing a mortality of more than fifty per cent. It has been already stated that not one of the Kroomen or blacks had suffered from an attack.—*Dublin Med. Press.*

